# The combination of the NS5A and cyclophilin inhibitors results in an additive anti-HCV inhibition in humanized mice without development of resistance

**DOI:** 10.1371/journal.pone.0251934

**Published:** 2021-05-20

**Authors:** Michael Bobardt, Christina M. Ramirez, Marc M. Baum, Daren Ure, Robert Foster, Philippe A. Gallay

**Affiliations:** 1 Department of Immunology & Microbiology, The Scripps Research Institute, La Jolla, California, United States of America; 2 Los Angeles (UCLA) Fielding School of Public Health, University of California, Center for Health Sciences, Los Angeles, CA, United States of America; 3 Department of Chemistry, Oak Crest Institute of Science, Monrovia, CA, United States of America; 4 Hepion Pharmaceuticals, Edison, New Jersey; National Institute of Infectious Diseases, JAPAN

## Abstract

We and others previously reported that the direct-acting agents (DAA) NS5A inhibitors (NS5Ai) and the host-targeting agents cyclophilin inhibitors (CypIs) inhibit HCV replication *in vitro*. In this study, we investigated whether the combination of NS5Ai and CypI offers a potent anti-HCV effect *in vivo*. A single administration of NS5Ai or CypI alone to HCV-infected humanized-mice inhibits HCV replication. The combination of NS5Ai with CypI suppresses HCV (GT1a, GT2a, GT3a and GT4a) replication in an additive manner. NS5Ai/CypI combinations provide a statistically more profound anti-HCV inhibition for GT2a and GT3a than GT1a and GT4a, leading to a fastest and deepest inhibition of GT2a and GT3a replications. Combining CypI with NS5Ai prevents the viral rebound normally observed in mice treated with NS5Ai alone. Results were confirmed in mice implanted with human hepatocytes from different donors. Therefore, the combination of NS5Ai with CypI may serve as a regimen for the treatment of HCV patients with specific genotypes and disorder conditions, which diminish sustain viral response levels to DAA, such as GT3a infection, cirrhosis, and DAA resistance associated with the selection of resistance-associated substitutions present at baseline or are acquired during treatment.

## Introduction

Approximately 71 million people worldwide are infected with HCV [1–3]. In HCV patients, the liver damage varies from negligible injuries to pronounced fibrosis, cirrhosis, or HCC. Worldwide deaths associated with HCV-induced complications are estimated to be ~600,000 [4]. Anti-HCV treatments have progressed profoundly during the last 20 years. Current HCV treatments includes highly successful combinations of pangenotypic direct-acting antivirals (DAA) with short period treatments (8–12 weeks), high sustained virological response (SVR) (>95%), and minimal side effects. However, the combination of specific viral genotypes (GT) and disorder conditions diminishes SVR levels (90–95%) to DAA such as HCV genotype 3 (GT3) infection, cirrhosis, and DAA resistance associated with the selection of resistance-associated substitutions (RASs) present at baseline or are acquired during treatment [4].

An option to avoid the retreatment of HCV patients for viral resistance and GT3 infection would be to incorporate into current FDA-approved DAA regimens, antivirals with high barrier to resistance and with different antiviral mechanisms of action. Such a class of antivirals are the cyclophilin inhibitors (CypI). The CypI non-immunosuppressive cyclosporine A (CsA) analogs–Alisporivir and CRV431 display high anti-HCV efficacy both *in vitro* and *in vivo* [5–12]. CypI neutralize the peptidyl-prolyl *cis-trans* isomerase activity of cyclophilin members by binding to their enzymatic hydrophobic pocket [13–16]. Alisporivir and CRV431 inhibit HCV infection by preventing the formation of complexes between host cyclophilin A (CypA) and HCV NS5A, leading to the inhibition of double membrane vesicles (DMVs) that normally serves as sanctuaries for efficient viral RNA replication [17–21]. Interestingly, NS5Ai like CypI also inhibit the formation of DMVs leading to an abortive infection [18]. In the present study, we compared the antiviral efficacy of NS5Ai and CypI used individually or in combination in HCV-infected humanized mice.

## Materials and methods

### Drugs

CRV431 was manufactured by chemical modification of cyclosporin A. Alisporivir, Velpatasvir, Ledipasvir and Sofosbuvir were purchased from MedChemExpress. Their purity exceeded 95% as determined by HPLC.

### Animal care

#### Animal housing

Individually ventilated cage (IVC) racks are employed to house our mice. HEPA-filtered air is delivered into solid bottom cages at a rate of 60 air changes/hour. Static mouse cages and IVCs are changed every week and every two weeks, respectively. Room environment: air conditioning, ventilation and heating functioning is routinely checked in case of system repairs and facility renovations at least once every 3 years. All animal rooms are equipped with a high/low thermo-hygrometer and its computerized controlled thermostat. The Department Animal Resources (DAR) staff check daily and record on the room activity log high/low temperatures and humidity of each animal room. Temperature settings are in agreement with Guide recommendations and are adjusted by the Engineering Department. Alarm points are fixed at ± 4˚F. High/low temperature alarms are communicated to the engineer on duty daily. DAR management is warned of excursions. Animal facilities are equipped with an Edstrom Industries Watchdog environmental monitoring system together with the automated building management system (BMS). The Watchdog system registers humidity, temperature and alarms sent to DAR personnel. Humidity levels are reliably maintained between 30–70% most of the year.

#### Diet

Food (Teklad LM-485 autoclavable diet) is given *ad libitum* to mice in wirebar lids. Water: our vivarium in the Department of Immunology & Microbiology is equipped with a reverse osmosis (R/O) water purification system and automatic watering distribution system from Edstrom Industries. DAR gets water quality reports from the City of San Diego monthly. R/O purified water is checked daily during the workweek. Chlorine concentration, conductivity, temperature, and pH level will also be monitored. Automatic water delivery systems are scheduled for daily in-line flushing (room and rack distribution lines). Quick disconnect drinking valves are cleaned during each cage change or more often if needed. Preventive maintenance and system sanitation are performed by DAR.

#### Acclimation period

Mice remain up to 72 hours to acclimate into their new housing environment.

#### Animal suffering

To reduce suffering, surgical procedures are performed under anesthesia (1–4% isoflurane) together with ketamine **(**120 mg/kg) and xylazine (10 mg/kg) i.p. Mice are checked every 15 minutes for heart and respiratory rates if the surgical procedure requires more time. Animals are provided buprenorphine (0.05–2.5 mg/kg s.c.) for 6–12 hours followed by flunixine meglumine (2.5 mg/kg s.c.) as a postoperative analgesic for 48 hours post-implantation. Mice are observed 2, 6 and 24 hours post-surgery with daily monitoring. Mice receive acidified water supplemented with sulfamethoxazole (or sulfadiazine) with trimethoprim at a final concentration of 0.65–1.6 mg/mL to avoid opportunistic bacterial colonization.

MUP-uPA-SCID/Beige mice were kept by DAR in our vivarium in accordance with protocols approved by the TSRI Ethics Committee, the Institutional Animal Care and Use Committee (Protocol Number: 11–0015). This study was conducted under strict accordance with the recommendations in the Guide for the Care and Use of Laboratory Animals of the National Institutes of Health. All efforts were made to reduce suffering. Cervical dislocation is the method of sacrifice used for the experimental mice. A power calculation was used to determine the sample size (10 mice/group).

### HCV chimeric mouse study

Transgenic mice carrying the uPA gene driven by the major urinary protein promoter were crossed onto a SCID/Beige background (MUP-uPA-SCID/Beige) [22]. MUP-uPA-SCID/Beige mice are healthier than former Alb-uPA mice and importantly offer an prolonged window from age 4 to 12 months for human hepatocytes engraftment and infection with HCV derived from concentrated supernatant of HCV-replicating cell culture [22]. MUP-uPA-SCID/Beige mice (gift from Dr. A. Kumar) (4 months old) were transplanted with human hepatocytes (10^7^ cells/mouse) (gift from Dr. Geller) as described previously [22]. Fresh counted viable human hepatocytes were transplanted within 24 hours after isolation. A 1 cm skin incision was made in the upper left quadrant of the abdomen to locate the spleen. Human hepatocytes were injected intra-splenically. Incision was closed with Vetbond tissue adhesive (3M Animal Care Products, St. Paul, MN). Humanization of liver-transplanted mice was analyzed by collecting blood weekly for human albumin quantification by ELISA (Bethyl Laboratories) according to the manufacturer’s instructions. Humanized mice with >300 μg/mL of human albumin were selected and randomized into groups (n = 10 mice/group). uPA transgene expression triggers mouse liver damage, which is quickly substituted by clusters of implanted human hepatocytes as demonstrated by human albumin immunostaining [31]. MUP-uPA-SCID/Beige mice engrafted with human hepatocytes were infected intravenously (i.v.) with HCV-infected chimpanzee plasma (100 infectious doses (CID_50_)/mL of genotype 1a, 2a, 3a and 4a) (gift from Drs. Lanford and Farci) or from concentrated virus from infected Huh7.5.1 cells. Alisporivir was dissolved in 5% absolute ethanol, 5% Cremophor EL, and 90% sterile saline solution, CRV431 in polyethylene glycol 300 molecular weight (PEG-300), and Velpatasvir, Ledipasvir and Sofosbuvir in DMSO and subsequently in 95% sterile saline solution. Drugs were given once at 50 mg/kg by oral gavage at the designated time points. Blood was collected retro-orbitally at the designated time points.

### HCV RNA quantification by real-time reverse transcription PCR

HCV RNA in serum was isolated using an acid guanidinium-phenol-chloroform method. HCV RNA quantification was conducted by real-time reverse transcription PCR based on TaqMan chemistry as described [23]. It is important to note that we previously demonstrated that there is a direct correlation between viral loads in blood and in liver [12].

## Results

### Velpatasvir, Ledipasvir, CRV431 and Alisporivir individually inhibits HCV replication in humanized-liver mice

**Fig 1:**
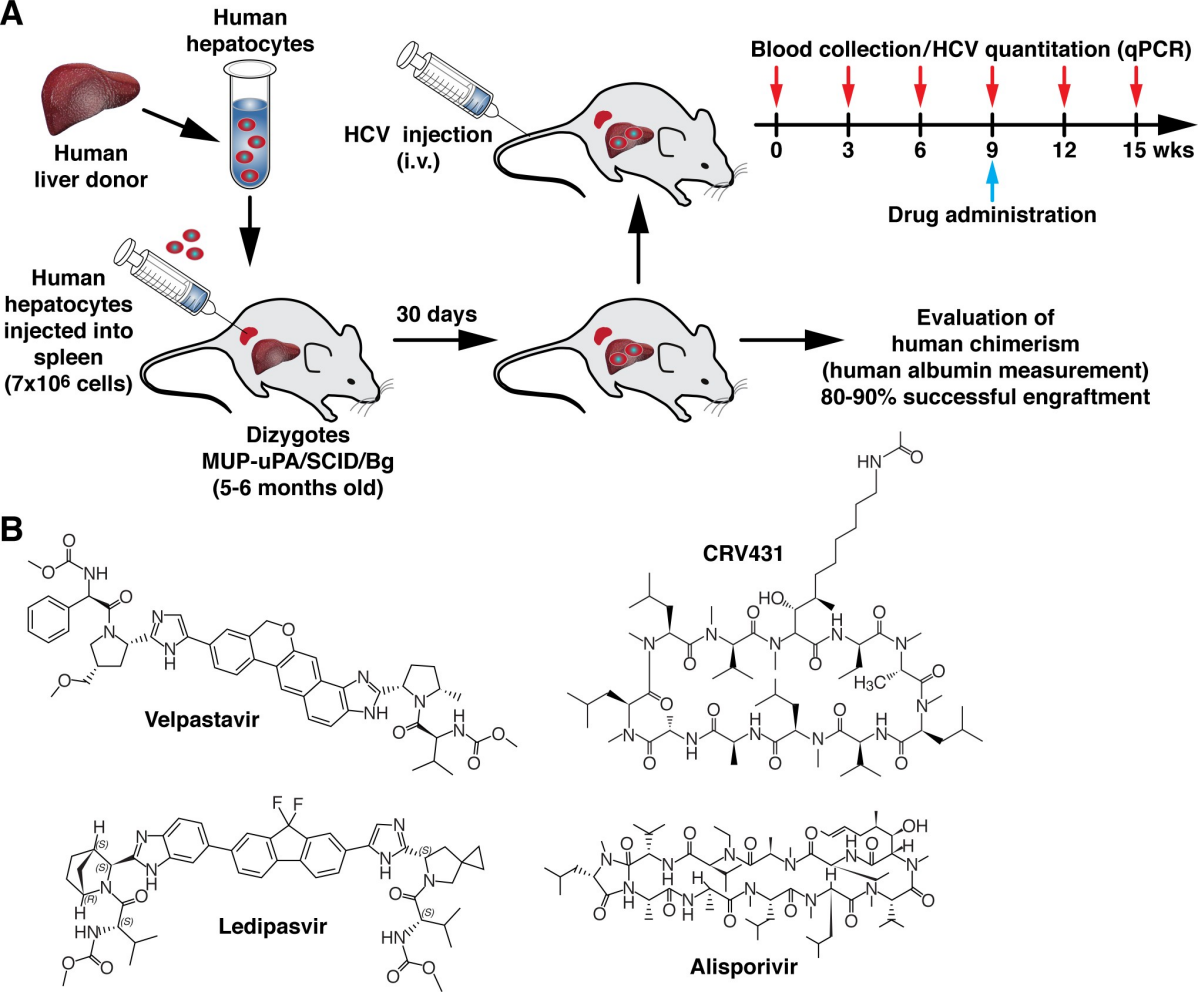
Humanized-liver mouse model for HCV infection. **A.** Experimental design for the creation and validation of the HCV infection model in humanized-liver chimeric MUP-uPA-SCID/Beige mice. **B.** Chemical structures of the NS5Ai Velpatasvir and Ledipasvir, and the CypI CRV431 and Alisporivir.

Previous studies including ours demonstrated that NS5i Velpatasvir and Ledipasvir as well as CypI CRV431 and Alisporivir [4,5,24,25] inhibit *in vitro* HCV infection from the majority of genotypes at a nM range. In this study, we initially asked whether these antivirals individually also suppress ongoing HCV replication *in vivo*. We took advantage of a humanized mouse model, which entails the implantation human hepatocytes into MUP-uPA-SCID/Beige mice (Fig 1) as originally developed by the Feinstone lab [22], and now developed in our lab [12]. After validating positive human hepatocyte engraftment by quantifying human albumin in blood (>300 ng/mL), HCV was given intravenously into mice. Viral loads were quantified by qPCR, and NS5Ai and CypI were given orally at the chosen time points.

**Fig 2:**
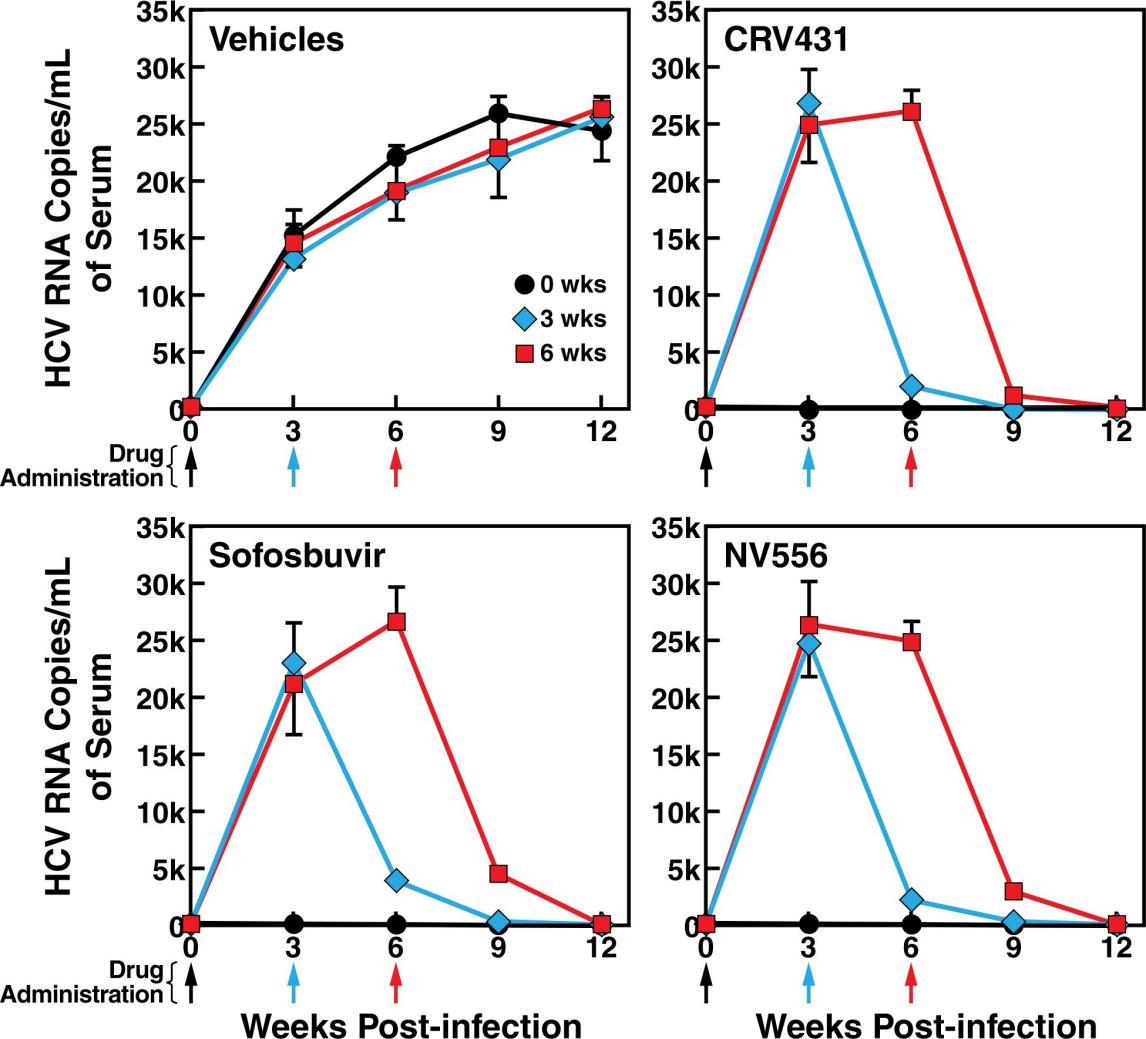
Individual analysis of anti-HCV efficacy of Velpatasvir, Ledipasvir, CRV431 and Alisporivir in humanized-liver chimeric mice. MUP-uPA-SCID-Beige mice implanted with human hepatocytes were infected i.v. with plasma from HCV-infected chimpanzee (100 infectious doses (CID_50_)/mL of GT1a, GT2a, GT3a and GT4a). Drugs (50 mg/kg) were given orally 9 weeks post-infection. Blood was collected retro-orbitally every week until week 15 post-HCV infection. Ten mice per treatment. Viral replication (HCV RNA copies/ml of serum) was quantified by real-time reverse transcription PCR. Error bars corresponds to the standard deviation (SD) between 10 animals. At week 12 and 15 post-infection, SD are small due to the minimal viral loads due to the drug treatment efficacy. Data are representative of two independent experiments.

A maximal HCV viremia replication (GT1a, GT2a, GT3a and GT4a) occurs approximately 9 weeks post-infection in vehicle-treated chimeric mice (Fig 2). Although the pattern of replication between genotypes is relatively similar, viral loads varies between genotypes in this particular chimeric mouse model (GT4a>GT1a>GT3a>GT2a). Note that we did not corroborate blood viral loads quantified by RT-PCR by liver immunohistochemistry (ICH) because it would require too large numbers of animals to be sacrificed at each time point, it would not be as quantitative as RT-PCR analyses routinely used in patients, and most importantly ICH would not provide additional information than RT-PCR analyses. We first tested the antiviral effect of NS5Ai and CypI used individually. Both NS5Ai Velpatasvir and Ledipasvir as well as both CypI CRV431 and Alisporivir efficiently suppress ongoing HCV replication of the four used genotypes when administered at the peak of viremia (9 weeks post-infection) (Fig 2). A full inhibition of viral replication by each NS5Ai and CypI used individually is achieved at approximately week 15 post-infection (Fig 2).

### The combination of NS5Ai with CypI provides additive inhibition of HCV replication in humanized-liver mice

**Fig 3:**
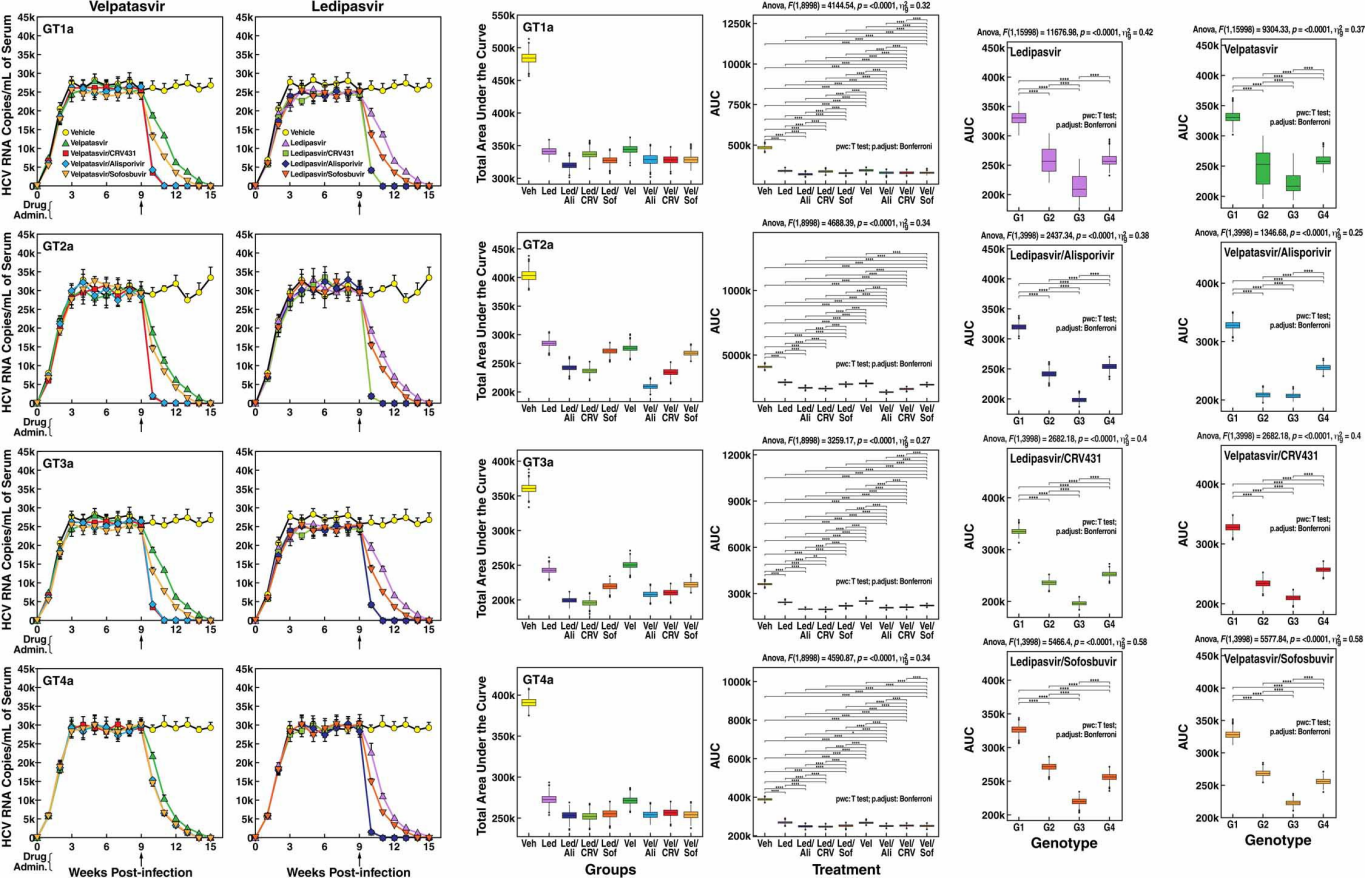
Combinational analysis of anti-HCV efficacy of Velpatasvir, Ledipasvir, Alisporivir and CRV431 in humanized-liver chimeric mice. **A.** MUP-uPA-SCID-Beige mice implanted with human hepatocytes were infected i.v. with plasma from HCV-infected chimpanzee (100 infectious doses (CID_50_)/mL of GT1a, GT2a, GT3a and GT4a). Drugs were orally given alone (50 mg/kg) or in combination (50/50 mg/kg) 9 weeks post-infection. Blood was collected until week 15 post-HCV infection. Ten mice per treatment. Viral replication (HCV RNA copies/ml of serum) was quantified by real-time reverse transcription PCR. Error bars corresponds to the SD between 10 animals. At week 12 and 15 post-infection, SD are small due to the minimal viral loads due to the drug treatment efficacy. **B.** Statistical analyses for drug combination effect between HCV genotypes. **C.** Statistical analyses for Ledipasvir combination effect on HCV GT1a, GT2a, GT3a and GT4a. **D.** Statistical analyses for Velpatasvir combination effect on HCV GT1a, GT2a, GT3a and GT4a. For each treatment/genotype combination, we simulated 1,000 draws using the mean and standard deviation from the experimental results to account for the uncertainty in estimating the Area Under the Curve for the level of viremia. Area Under the Curve was estimated using the trapezoidal method. Groups were compared using t-tests and the permutation test. P-values were adjusted for multiple comparisons using the Bonferroni correction. Data are representative of two independent experiments.

We next compared the potency of NS5Ai and CypI used in combination. Chimeric mice were infected as above and treated with NS5Ai Velpatasvir and Ledipasvir individually or in combination with the CypI CRV431 and Alisporivir 9 weeks post-infection. NS5Ai used individually display profiles of inhibition of viral replication for all four genotypes (Fig 3) similar to those observed above (Fig 2). In contrast, all combinations of NS5Ai with CypI—Velpatasvir/CRV431, Velpatasvir/Alisporivir, Ledipasvir/CRV431 or Ledipasvir/Alisporivir–provide additive antiviral inhibition for GT1a and GT4a (Fig 3). NS5Ai/CypI combinations, by reducing blood levels of HCV loads more efficiently than NS5Ai used alone, shorten the time period for full suppression of viral replication (Fig 3). Importantly, NS5Ai/CypI combinations provide a statistically more profound anti-HCV inhibition for GT2a and GT3a (Fig 3B–3D), leading to a fastest and deepest inhibition of GT2a and GT3a replications (from week 15 to 11) than those of GT1a and GT4a replications (from 15 weeks to 13) (Fig 3). Both Velpatasvir/Sofosbuvir and Ledipasvir/Sofosbuvir combinations also provide an additive inhibitory effect on all four genotypes (Fig 3). The combinations of NS5Ai with Sofosbuvir provide a less profound inhibition than the combinations of NS5Ai with CypI, at least on GT2a and GT3a (Fig 3). We obtained similar additive inhibitory profiles in two independent experiments using human hepatocytes from two donors, and different viral stocks of GT1a to GT4a. Human albumin blood levels remained constant over the full period of the experiment (15 weeks) and among all groups. Similarly, blood levels of human aspartate aminotransferase (AST) and alanine aminotransferase (ALT) remained constant among all groups over the full period of the experiment (15 weeks), suggesting that the integrity and functions of the chimeric livers are preserved.

### No evidence of HCV rebound after extended interruption of the treatment with a combination of NS5Ai with CypI

**Fig 4:**
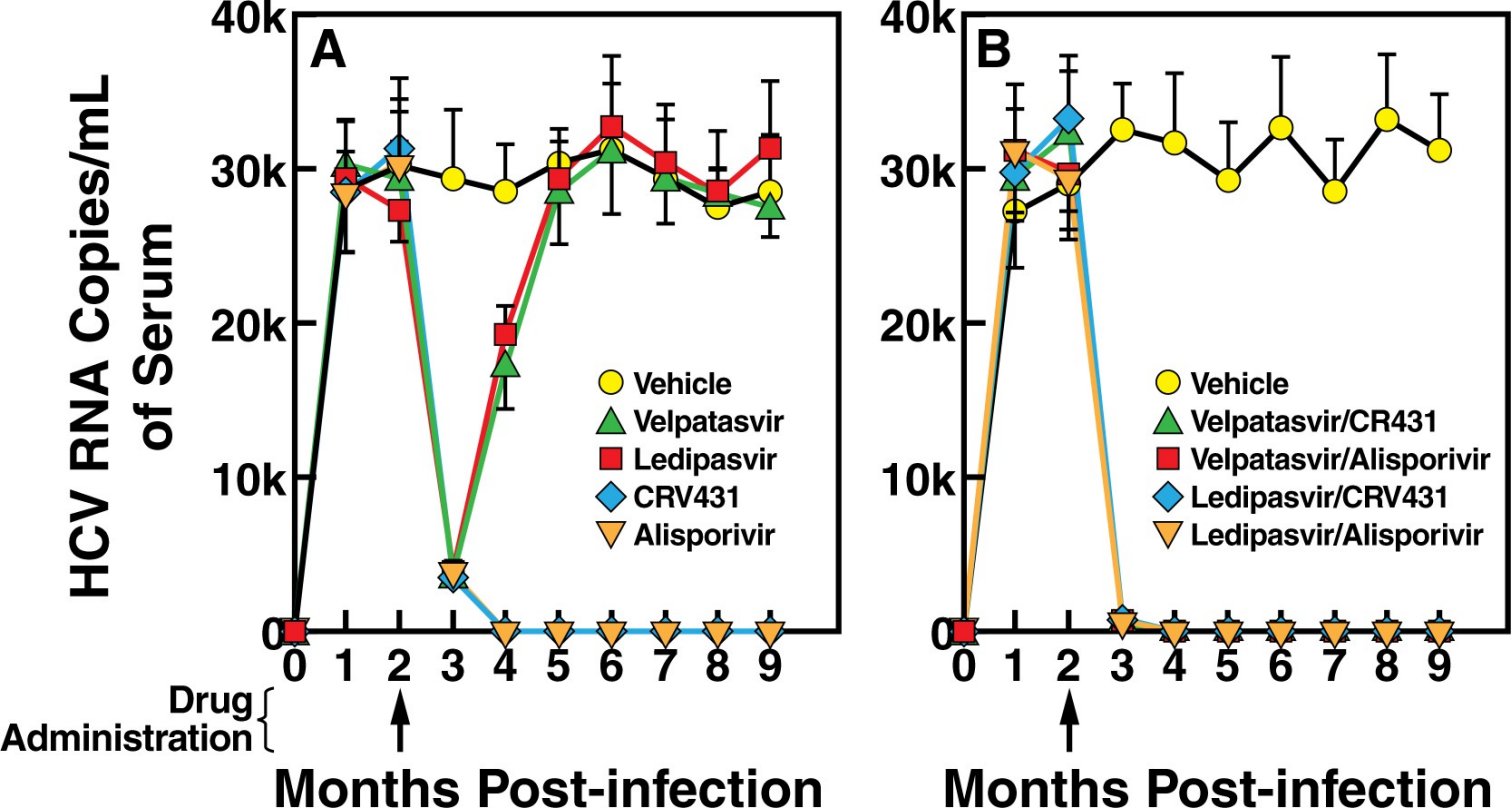
HCV rebound analysis in humanized-liver chimeric mice. MUP-uPA-SCID-Beige mice implanted with human hepatocytes were infected i.v. with plasma from HCV-infected chimpanzee (100 infectious doses (CID_50_)/mL of GT1a, GT2a, GT3a and GT4a). Drugs—Velpatasvir, Ledipasvir, CRV431, Alisporivir—(50 mg/kg) were orally given alone or in combination 2 months post-infection. Ten mice per treatment. Blood was collected until month 9 post-HCV infection. Viral replication (HCV RNA copies/ml of serum) was quantified by real-time reverse transcription PCR. At week 12 and 15 post-infection, SD are small due to the minimal viral loads due to the drug treatment efficacy. Data are representative of two independent experiments.

We then asked whether the combination of NS5Ai with CypI can prevent viral rebound after single dose drug combination administration. HCV-infected humanized mice received 2 months post-infection a single dose of Velpatasvir, Ledipasvir, CRV431, Alisporivir alone (Fig 4A) or in combinations—Velpatasvir/CRV431, Velpatasvir/Alisporivir, Ledipasvir/CRV431 or Ledipasvir/Alisporivir (50/50 mg/kg) (Fig 4B). Viral replication was analyzed monthly over a period of 9 months. In vehicle-treated chimeric mice, viral replication was relatively stable until 9 months post-infection (Fig 4). Viral rebounds were observed 4 to 5 months after the single dose drug treatment in NS5Ai Velpatasvir- and Ledipasvir-treated humanized mice (Fig 4A). In this chimeric mouse model, viral rebound was normally observed 9–10 weeks post-drug administration after a single dose of NS5Ai nine (Fig 2) or eight weeks (Fig 4) post-infection. In contrast, no viral rebound was observed in CypI CRV431- and Alisporivir-treated mice (Fig 4A). Together the data further suggest a low and high barrier to drug resistance by NS5Ai and CypI, respectively. No viral rebound was also observed for all NS5Ai/CypI combinations of NS5Ai with CypI—Velpatasvir/CRV431, Velpatasvir/Alisporivir, Ledipasvir/CRV431 and Ledipasvir/Alisporivir—9 months post-infection (Fig 4B). Altogether these data demonstrate that the combination of two classes of anti-HCV agents–the DAA NS5Ai and the HTA CypI–provide additive antiviral efficacy in HCV-infected in humanized mice, without drug resistance and viral rebound.

## Discussion

Present-day HCV therapies involves highly efficient regimens of pangenotypic DAA with relatively brief period treatments (8–12 weeks), >95% SVR, and minor adverse effects. Nevertheless, the association of particular HCV GT and illness conditions significantly reduces SVR levels. This includes the combination of HCV GT3, cirrhosis, and DAA resistance associated with the selection of RASs present at baseline or acquired during DAA treatment [3,4]. Considering this combination of disorder conditions, a significant percentage of HCV patients requires a retreatment. A majority of current HCV treatments include NS5Ai due to their unique property to rapidly and profoundly reduce viral loads. However, NS5Ai offer a low barrier of drug resistance due to resistance mutations in the NS5A protein either acquired during the DAA treatment or present at the baseline [3,4]. On the other hand, we and others showed that CypI offer a high barrier of resistance both in *vitro* and *in vivo* [5–8] likely due to the fact that CypI target a host protein–cyclophilin A–while NS5Ai target a viral protein—NS5A [26–28]. Moreover, NS5Ai bind to the domain I of NS5A [26–28], and cyclophilin A binds to the domain II of NS5A. CypI, by binding directly to the peptidyl-prolyl *cis-trans* isomerase pocket of cyclophilin A, block the contact between the host protein and the domain II of NS5A [29–32]. One can envision that a combination of NS5Ai and CypI, which target two distinct domains of NS5A, renders more challenging for the virus to develop mutations required for drug resistance. To date, no cross-resistance has been observed between CypI and NS5Ai, at least *in vitro* [33]. Specifically, CypI suppress replication of NS5Ai-resistant replicons, and NS5Ai suppress the replication of the CypI-resistant replicon [5]. Since NS5Ai monotherapy leads to fast viral breakthrough due to mutations in the domain I of NS5A [33–36], the absence of cross-resistance under dual CypI with NS5Ai drug pressure is highly relevant. A combination of NS5Ai with CypI should reduce the rate of emergence of NS5Ai-mediated viral breakthroughs as reflected by our observation of an absence of viral rebound in HCV-infected mice treated with combinations of NS5Ai and CypI. We reported that the D320E substitution in the domain II of NS5A occurs during CypI selection [30], however this amino acid substitution only marginally attenuates HCV vulnerability to CypI and does not allow drug resistance or viral breakthrough [31].

In the present study, we investigated whether the combinations of NS5Ai with CypI can provide *in vivo* potent pangenotypic suppression of HCV replication without emergence of resistance in humanized mice.

First, we demonstrated that the NS5Ai Velpatasvir and Ledipasvir as well as the CypI CRV431 and Alisporivir individually suppress HCV replication in humanized-liver mice. It is interesting to note that a single dose of a high concentration (50 mg/kg) of chosen anti-HCV drugs administered 9 weeks post-infection was sufficient to prevent rapid re-infection 6 weeks post-drug administration. This is likely due to their high antiviral potency and their extended half-life in the body (CRV431 ~25h, Alisporivir ~25h, Velpatasvir ~15h, Ledipasvir ~47h and Sofosbuvir ~27h). Note that we are perfectly aware that the drug treatment used in this study does not mimic the DAA daily treatment in HCV patients. Our goal was to test the efficacy of a single dose of drug administration (CypI alone, NS5Ai alone or in combination) in terms of viral replication suppression and viral rebound. Since we previously demonstrated the antiviral efficacy of CypI alone in the chimeric mouse model [12], we only present in the present study the antiviral efficacy of NS5Ai alone or in combination with CypI. It is also important to emphasize that we and others already validated a successful HCV infection of engrafted humanized liver tissue [12,22]. Since HCV cannot infect mice, our observation that a robust viral replication occurs in MUP-uPA-SCID/Beige mice implanted with human hepatocytes, it provides another validation of a successful human hepatocyte engraftment.

Second, we showed that the combination of NS5Ai with CypI offers an additive inhibition of HCV replication in humanized mice. The combination of NS5Ai with CypI provides statistically a more profound inhibitory effect on GT2a and GT3a than GT1a and GT4a. The fact that NS5Ai and CypI target two distinct domains of NS5A may explain that their combination inhibits efficiently all genotypes tested. The combined action of NS5Ai and CypI may hamper NS5A binding to the viral RNA. Since the domain I of NS5A encompasses the viral RNA binding region [37,38], NS5Ai, by binding to this domain, may diminish NS5A-viral RNA interactions. The Harris lab obtained convincing data showing that cyclophilin A promotes NS5A binding to the viral RNA, and that CypI prevent this effect [39]. Therefore, the combination of NS5Ai with CypI may cause additive or synergistic inhibition of the binding of NS5A to the viral RNA, resulting in the inhibition of viral RNA replication and/or HCV replication complexes machinery. One can envision that either the affinity of cyclophilin A to the domain II and/or the affinity of the viral RNA to the domain I of GT2 and GT3 differs from that of GT1 and GT4, and that the affinity of CypI and NS5Ai to domains II and I, respectively, differs between genotypes, explaining the antiviral difference in sensitivity between GT2/GT3 and GT1/4. Furthermore, one can envision that GT2 and GT3 rely more on the protection and formation of double membrane vesicles in the ER where HCV replicates and that both NS5Ai and CypI disrupt [17–20]. Moreover, the combinations of NS5Ai with CypI provides a superior inhibitory effect on GT2a and GT3a than the combinations of NS5Ai with Sofosbuvir. Our observation of a high additive anti-HCV effect by the combination of NS5Ai with CypI may represent an important therapeutic tool for the difficult to treat GT3. These *in vivo* data are in accordance with our previous work showing that the CypI Alisporivir and NS5Ai daclatasvir combination offers pangenotypic additive to synergistic inhibition of HCV infection *in vitro* in the absence of cross-resistance. In order to calculate an index to determine whether drug combinations exert an additive effect or more interestingly, a synergistic effect, multiple drug concentrations should be tested over time in a set ratio, starting with very low, low, EC_50_, high, very high for each drug alone and then in combination. However, in the present *in vivo* study, due to statistically necessary high numbers of animals per treatment, we were unable to determine whether the combinations of NS5Ai with CypI exert a synergistic effect on GT1a, GT2a, GT3a and GT4a since we used a single drug concentration (30 mg/kg).

This study presents the first *in vivo* demonstration that the combinations of the DAA NS5Ai with the HTA CypI provide additive inhibitory effects on multiple HCV strains (GT1a to GT4a). Our *in vivo* data demonstrate that the combination of NS5Ai with CypI represents a scientifically supported therapeutic combination based on their distinct mechanisms of action on NS5A. NS5Ai, which have a low barrier to resistance and rapid viral load decline in patients by targeting the viral protein NS5A, represent ideal drug partners for CypI, which are pangenotypic, have a high barrier to HCV resistance and target a host protein. Thus, the combination of NS5Ai with CypI presents an attractive opportunity for future HCV therapy as an oral IFN-free regimen. Importantly to this study, we recently demonstrated that CypI provide major beneficial therapeutic effects against the development of liver damage. We reported that CypI CRV431, Alisporivir or NV556 treatments alone significantly reduce both liver fibrosis and hepatocellular carcinoma in multiple nonalcoholic steatohepatitis (NASH) and nonalcoholic fatty liver disease (NAFLD) mouse models [40–42]. Our demonstration of the dual therapeutic effects of CypI that consist i) of suppressing efficiently HCV replication in an additive and synergistic effect when combined with NS5Ai, and ii) of preventing the deleterious development of liver fibrosis and liver cancer, reveals CypI as an interesting class of anti-HCV and anti-liver damage agents to be included into current DAA treatment to treat chronic hepatitis C and HCV-induced liver fibrosis and hepatocellular carcinoma.
